# The value of multiparametric MRI radiomics in predicting IDH genotype in glioma before surgery

**DOI:** 10.3389/fonc.2023.1265672

**Published:** 2023-11-27

**Authors:** Yuanzi Liang, Wenjuan Liu, Dong Bai, Junqi Hu, Zhiqun Wang

**Affiliations:** ^1^Department of Radiology, Aerospace Center Hospital, Beijing, China; ^2^Department of Nephrology, Aerospace Center Hospital, Beijing, China

**Keywords:** glioma, isocitrate dehydrogenase, radiomics, magnetic resonance imaging, multiparametric

## Abstract

**Objective:**

To explore the value of multiparametric magnetic resonance imaging(MRI) radiomics in the preoperative prediction of isocitrate dehydrogenase (IDH) genotype for gliomas

**Methods:**

The preoperative routine MRI sequences of 114 patients with pathologically confirmed grade II-IV gliomas were retrospectively analysed. All patients were randomly divided into training cohort(n=79) and validation cohort(n=35) in the ratio of 7:3. After feature extraction, we eliminated covariance by calculating the linear correlation coefficients between features, and then identified the best features using the F-test. The Logistic regression was used to build the radiomics model and the clinical model, and to build the combined model. Assessment of these models by subject operating characteristic (ROC) curves, area under the curve (AUC), sensitivity and specificity.

**Results:**

The multiparametric radiomics model was built by eight selected radiomics features and yielded AUC values of 0.974 and 0.872 in the training and validation cohorts, which outperformed the conventional models. After incorporating the clinical model, the combined model outperformed the radiomics model, with AUCs of 0.963 and 0.892 for the training and validation cohorts.

**Conclusion:**

Radiomic models based on multiparametric MRI sequences could help to predict glioma IDH genotype before surgery.

## Introduction

Gliomas are the most common primary brain tumors that originate from neuroepithelial cells and can occur in any part of the central nervous system(CNS) (1). Previously, World Health Organization(WHO) classified gliomas into grades I-IV, with grades I and II considered low-grade gliomas (LGG) and grades III and IV considered high-grade gliomas (HGG) (2), with a median survival of 14 months for glioblastomas (grade IV) and more than 7 years for grades II and III gliomas (3, 4). However, many studies have reported that the prognosis of gliomas is not related to the pathological grade, but is mainly based on the molecular characteristics of the tumor, and if the same genotype exists, similar biological behavior and prognosis may exist even if the tumors have different pathological grades (5, 6). The 2016 WHO CNS tumor classification included molecular features, particularly isocitrate dehydrogenase (IDH), which is divided into IDH mutant(IDH-M)and IDH wild type(IDH-W),on a histological basis for the first time and used it as one of the important bases for molecular typing of gliomas (7, 8). Low-grade gliomas with IDH-W are similar to glioblastomas in terms of molecular features and prognosis, while IDH-M gliomas have a better prognosis than IDH-W (9, 10). The impact of total tumor resection on the prognosis of low-grade gliomas has been reported to depend on IDH mutation status (11). Therefore, preoperative prediction of IDH status is necessary for appropriate treatment planning.

Currently, IDH genotypes are identified mainly by sequencing or immunohistochemistry of tumor specimens, which can only be obtained after surgery, and even biopsies of unresectable gliomas carry the risk of neurological impairment, and the small samples obtained do not reflect the full heterogeneity of the entire tumor (6, 12, 13). To overcome these limitations, there is an urgent need to establish a non-invasive technique to identify the IDH genotype of the tumor (14, 15), thus MRI examination is of great value in the preoperative diagnosis of glioma. At this stage, studies have evaluated the performance of various machine learning algorithms in predicting glioma genotypes (16–20). High-throughput features from MRI have been shown to be highly advantageous and effective in predicting the classification of IDH (15). Conventional MRI examinations correlate with IDH genotype and its prognosis by tumor morphology, border and enhancement, but most of them rely on the subjective diagnosis of radiologists and cannot be analyzed comprehensively from the whole tumor area. Radiomics can extract a large number of intrinsic features that cannot be observed by the naked eye and analyze the shape and texture of images (14, 21–23), which shows great advantages and values in the diagnosis of glioma.

In recent years, studies on preoperative prediction of tumor genotype by radiomics have been widely carried out, and the methods and results of different studies are not the same. Zhang et al. (18) predicted IDH-M in LGGs preoperatively by multiparametric MRI radiomics model and obtained AUC value of 0.83, with T2-weighted imaging(T2WI) images being the most important.Another study titled “Predicting IDH Mutation Status in Low-Grade Gliomas Based on Optimal Radiomic Features Combined with Multi-Sequence Magnetic Resonance Imaging, 2022 (24)” concluded that a multiparametric radiomics model of T2-weighted-fluid-attenuated inversion recovery (T2-FLAIR) is most effective in distinguishing IDH mutation status in low-grade gliomas. However, Niu et al. (20) found that contrast-enhanced T1-weighted imaging (CE-T1WI) radiomics model could effectively predict IDH genotype in high-grade glioma, which is inconsistent with the results of the the two aforementioned studies. Therefore, despite numerous studies on using radiomics models to predict the IDH genotype status of gliomas, the research results are not conclusive and still exhibit certain differences. Further research from more clinical centers is required to enhance the accuracy of the model’s results. Sun et al (25) concluded that a combined machine learning algorithm exhibits excellent predictive performance in non-invasively predicting the molecular subtypes of lower-grade glioma (LGG) preoperatively. Several studies have incorporated clinical data into radiomics to build a combined model and found superior results (24–27). Zhou et al (28) and Tan et al (27). concluded that incorporating age information can improve the predictive results of the models. Furthermore, compared to imaging features, age information has a higher predictive value. This finding is of great importance for clinical practice, as clinical information can be obtained preoperatively and can provide more valuable information for treatment and prognosis. Therefore, more research results are needed to corroborate this conclusion. Furthermore, several studies have integrated functional sequences like perfusion⁃weighted imaging(PWI) and diffusion tensor imaging(DTI) to develop radiomics models for the prediction of IDH gene status in gliomas (29, 30). These studies have achieved satisfactory outcomes. However, a meta-analysis (1) indicates that despite the growing adoption of advanced imaging sequences for constructing feature models, traditional MRI sequences exhibit superior specificity in predicting IDH gene status in gliomas.Currently, most research is limited to studying low-grade or high-grade gliomas, which are limited by pathological findings and have variable results.

In this study, all high-grade and low-grade gliomas were included and constructed radiomics models based on multiparametric MRI sequences, including T1WI, T2-FLAIR, CE-T1WI, and apparent diffusion coefficient (ADC).Additionally, a combined model of radiomics features and clinical data has been established, making the model results more stable and enabling a more comprehensive prediction of the applicative value of the IDH genotype in gliomas.

## Materials and methods

### Patients

**Figure 1:**
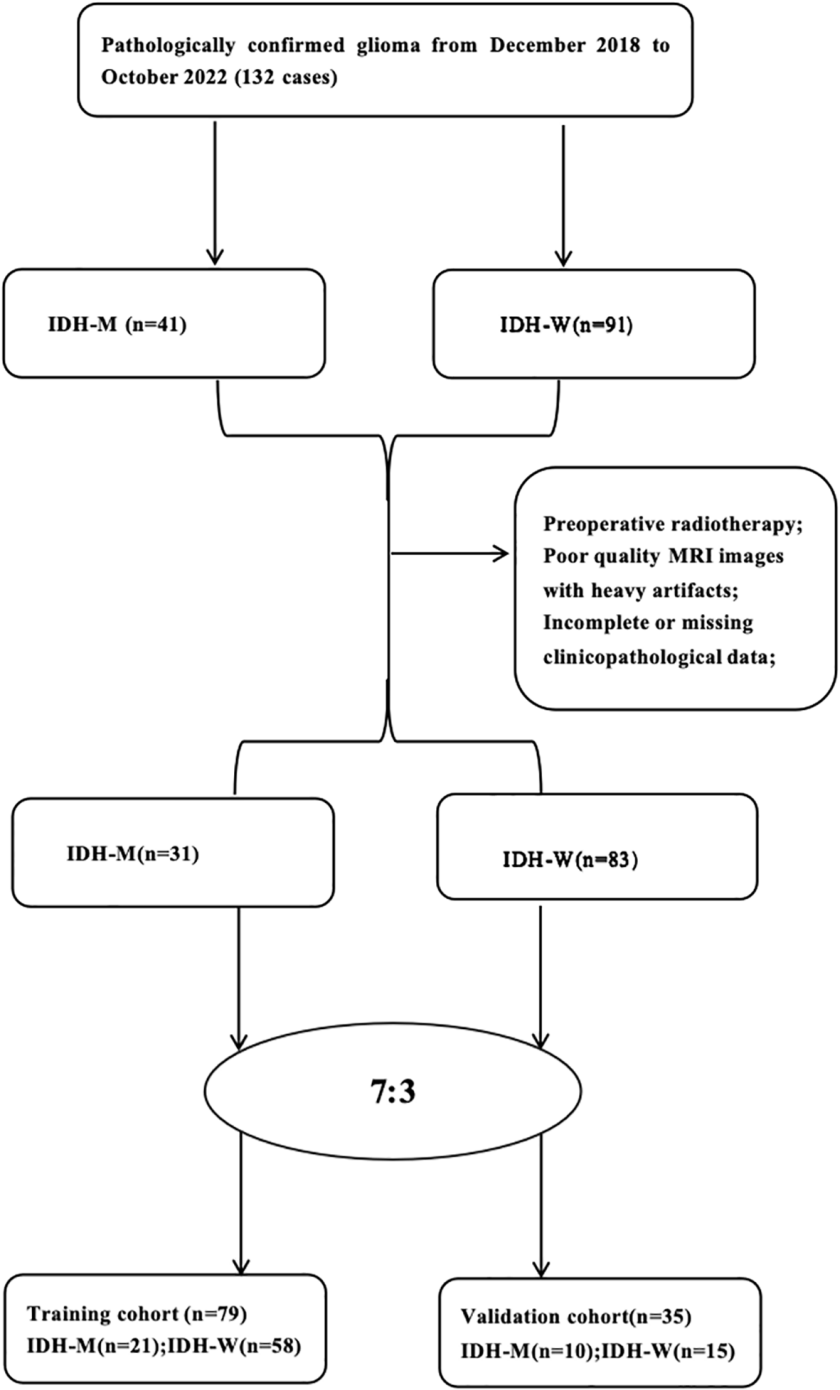
Flow diagram of the study population.

Clinical and radiological data of 132 patients with glioma who underwent preoperative MRI at the Aerospace Center Hospital from December 2018 to October 2022 were retrospectively collected. According to WHO classification of central nervous system tumors, the pathological findings were grade II-IV glioma. Inclusion criteria were the following: (1) pathological data reported as glioma; (2) preoperative cranial MRI examination; (3)Patients over 18 years old;(4) complete clinical data; (5) no history of other brain tumors. Exclusion criteria: (1) preoperative radiotherapy; (2) poor quality MRI images with heavy artifacts; (3) incomplete or missing clinicopathological data. The study finally included 114 patients with glioma, including 64 males and 50 females. Among the 114 patients, 83 were IDH-W and 31 were IDH-M. Clinical information of patients was collected, including age, gender, pathological grade of glioma, whether peritumoral edema, whether necrosis was present in the tumor, whether the tumor was enhancing, and location of the lesion. Patients were randomly divided into training and validation cohort according to the ratio of 7:3. A flow diagram of patients is shown in **Figure 1**. This paper is a retrospective study, which was approved by our ethical committee.

### MRI acquisition

MRI examination was performed using a 3.0T MRI scanner (skyra, Simens, Germany) and an 8-channel phased-array coil. The patient was examined in the supine position in head advanced scanning mode. Conventional MRI scan sequence and parameters: T1WI: Repetition time(TR)=1800ms, Echo time(TE)=8.5ms, Field of view(FOV)= 240×240mm^2^,matrix=256×256, layer thickness=5mm; T2WI: TR=4000ms, TE=94ms, matrix=256×256, FOV=240×240mm^2^, layer thickness=5mm; Contrast-enhanced T1-weighted imaging(CE-T1WI):TR=1800ms, TE=9ms,FOV=240×240mm^2^, matrix= 320×320,layer thickness 5mm. The contrast agent was gadopentetate glucosamine at a dose of 0.1 mmol/kg and a flow rate of 2.0-3.0 ml/s. The DWI sequence was implemented with *b* values of 0 and 1000 mm ^2^/s. TR=4300ms, TE=64ms, matrix= 164×164, FOV=240× 240mm^2^; layer thickness 6 mm. Apparent diffusion coefficient(ADC) maps were performed on the the workstation was generated automatically by DWI.

### Data preprocessing and ROI segmentation

**Figure 2:**
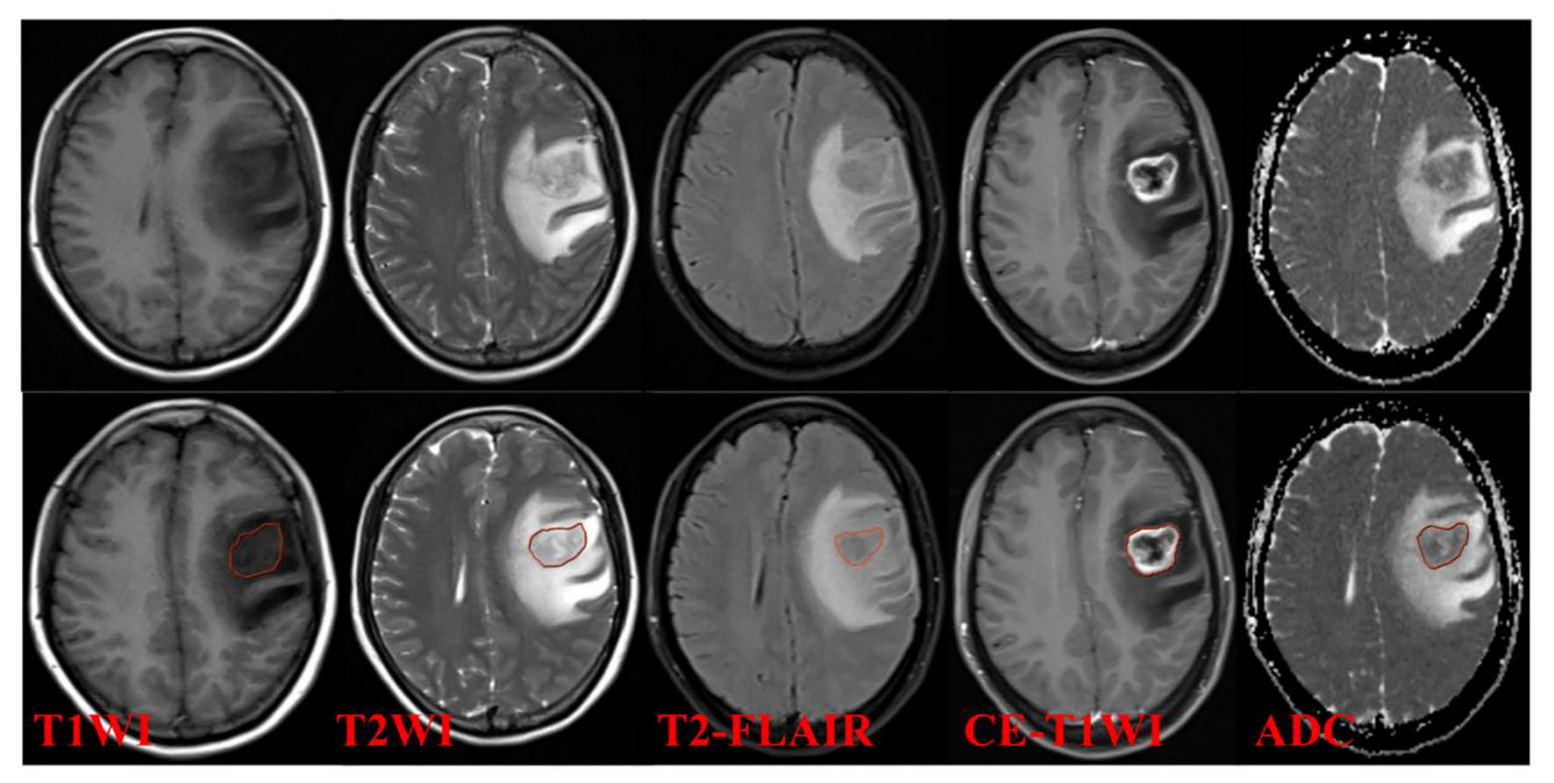
Examples of ROI segmentation.

The images were analyzed separately and independently by two radiologists with 3 years of experience in neurological MRI diagnosis using a double-blind method, and each tumor was manually outlined layer by layer, and the region of interest (ROI) of the entire tumor was manually mapped using the Deepwise Multimodal Research Platform version 2.2 (https://keyan.deepwise.com, Beijing Deepwise & League of PHD Technology Co., Ltd, Beijing, China.). The outline included tumor enhancement and areas of necrosis and cystic changes, but not peritumoral edema (**Figure 2**). Two radiologists with 3 years of experience in cranial MRI diagnostics simultaneously outlined regions of interest and extracted features for interclass correlation coefficients (ICC). One of the radiologists outlined and extracted features again after 2 weeks and compared them with the first features to evaluate the concordance of imaging histology features within the group. features with ICC > 0.75 were considered to have better concordance.

### Feature extraction

All images were resampled to 2 mm×2 mm×2 mm for the same resolution, and the intensity of them were scaled to 0-100 before radiomics feature extraction. For feature extraction, a total of 10 image filtering methods were applied to the images. The specific details of these filtering methods can be found in **Supplementary Table S1**. These methods involved mathematical processing techniques such as Laplacian of Gaussian (LoG) filtering, wavelet filtering, gradient calculation, Local Binary Patterns (LBP) in both 2D and 3D, as well as non-linear intensity transformations like square, square root, logarithm, and exponential.It is important to note that the features were not only extracted from the original image but also from the images subjected to the aforementioned preprocessing steps.

The features we analyzed in this study included the first-order features, the shape features. The texture features included the gray-level co-occurrence matrix (GLCM), gray-level run-length matrix (GLRLM), gray-level size zone matrix (GLSZM), and gray-level dependence matrix (GLDM). These features capture various aspects of the image texture, providing information about the spatial relationships and patterns within the image (21, 31–33).

Overall, a total of 1906 radiomics features were extracted for each lesion in the study, including features derived from both the original and filtered images. These features offer a comprehensive representation of the lesion characteristics, potentially enabling more accurate and detailed analysis in the context of the study.

### Feature selection and radiomics model construction

Different features have different means and variances, which can vary widely; we performed Z-score normalization, and after normalization, Z now equals 0 and STD equals 1, making all features comparable. The features were then analyzed by the Mann-Whitney U-test, and features with two types of differences (P<0.05) were retained. To eliminate severe covariance, the linear correlation coefficient ρ between features was first calculated, and one of the features was removed when ρ ≥ 0.75 until the linear correlation coefficient between all feature pairs was less than 0.75. The extracted features are reduced and transformed using principal component analysis (PCA). The PCA features obtained after conversion retain the most important information in the original features, alleviate noise and redundant information interference to a certain extent, and eliminate the influence of the original features on each other. On this basis, feature selection was performed for each feature and label pair using the F-test, and all features were ranked by histological grading to calculate individual F-values, and selection based on this ranking ensured that the most informative ones could be selected. Finally, multi-factor logistic regression analysis was performed to build a radiomics model.

Logistic regression model was used to build the prediction model of IDH. The area under the curve (AUC) of the receiver operating characteristic (ROC) was used to evaluate the diagnostic efficiency of the model (0.5 < AUC < 0.7 for low diagnostic efficiency, 0.7 < AUC < 0.9 for moderate diagnostic efficiency, and 0.9 < AUC for high diagnostic efficiency), and calculate their sensitivity, specificity and accuracy. Calibration curves were plotted to analyze the model calibration efficacy.

### Clinical model construction

Clinical characteristics were studied for gender, grading, and age. Radiologists with 15 years of experience were evaluated for imaging features including tumor border (well or ill), cystic necrosis (yes, no), peritumor edema (yes, no), tumor enhancement, tumor site (frontal, occipital, parietal, temporal, central, cerebellum,two or more), and univariate analysis was performed to identify potential clinico-radiological differences between the IDH-M and IDH-W groups in the training and validation cohorts that were significantly different characteristics (**Table 1**). A multifactorial logistic regression approach was used to build clinical model.

**Table 1:** Clinical and radiological characteristics of patients.

	Training cohort (n=79)		P	Validation cohort (n=35)		P
	IDH-W (n=58)	IDH-M (n=21)		IDH-W (n=25)	IDH-M (n=10)	
Gender			0.504			0.829
Male	31 (53%)	13 (60%)		14 (52.9%)	6 (66.7%)	
Female	27 (47%)	8 (40%)		11 (47.1%)	4 (33.3%)	
Age (years)	59.7 ± 15.8	44.4 ± 11.8	<0.001	56.6 ± 19.5	40.3 ± 13.5	<0.001
Grade			0.873			0.350
II	12 (21.2%)	4 (24%)		4 (17.6%)	3 (33.3%)	
III	14 (25.8%)	7 (32%)		5 (17.6%)	2 (16.7%)	
IV	32 (53.0%)	10 (44%)		16 (64.7%)	5 (50%)	
Peritumoral edema			0.304			0.867
yes	52 (87.9%)	17 (84%)		22 (82.4%)	9 (83.3%)	
no	6 (12.1%)	4 (16%)		3 (17.6%)	1 (16.7%)	
Cystic and necrosis			0.924			0.714
yes	38 (62.1%)	14 (56%)		19 (76.5%)	7 (66.7%)	
no	20 (37.9%)	7 (44%)		6 (23.5%)	3 (33.3%)	
Enhancement			0.02*			0.09
yes	56 (95.5%)	17 (80%)		23 (94.1%)	7 (100%)	
no	2 (4.5%)	4 (20%)		2 (5.9%)	3 (0%)	
Tumor location			0.742			0.524
Frontal lobe	11 (18.2%)	7 (40%)		5 (17.6%)	3 ( (33.3%))	
Temporal lobe	12 (19.7%)	5 (16%)		4 (11.8%)	2 (16.7%)	
Occipital lobe	10 (15.2%)	2 (8.0%)		3 (11.8%)	2 (16.7%)	
Parietal lobe	1 (1.5%)	0 (0%)		0 (0%)	0 (0%)	
Central area	15 (31.8%	4 (20%)		10 (41.2%)	3 (50%)	
Cerebellum	2 (3.0%)	0 (0%)		1 (5.9%)	0 (0%)	
Two or more	7 (10.6%)	3 (16%)		2 (11.8%)	0 (0%)	

### Statistical analysis

The IBM SPSS 25.0 (https://www.ibm.com) software was used, and comparisons of measures that conformed to a normal distribution with homogeneous variances were performed using the independent samples t test, otherwise the Mann-Whiney U test was used. Count data were analyzed by chi-square test, and P < 0.05 was considered a statistically significant difference.

## Results

### Clinicopathological data

A total of 114 patients were finally enrolled and randomly assigned to the training cohort (n = 79) and validation cohort (n = 35) in this study. There were 64 males and 50 females with an average age of 50.3 ± 15.2 years. The clinical data of the patients are shown in **Table 1**. Among all clinical characteristics, age was the count data, which was tested to be not normally distributed, so the Mann-Whiney U test was used. The rest of the characteristics were measures and the chi-square test was used. The results showed that age and whether the tumor was enhanced in the clinical data were statistically significant (P < 0.05); the differences in gender, peritumoral edema, lesion site, and tumor necrosis between the two groups were not statistically significant (P > 0.05).

### Radiomics feature selection

The mean ICC of these features was 0.792 (95% CI 0.678 to 0.883), showing good interobserver agreement. We extracted 1906 features from each sequence (396 first-order features, 14 shape features and 1496 texture features including 484 Gray Level Co-occurence Matrix (GLCM), 352 Gray Level Run-Length Matrix (GLRLM), 352 Gray Level Size Zone Matrix (GLSZM), and 308 Gray Level Dependence Matrix (GLDM). In total, 7624 radiomics features were extracted from four MRI single sequences for each patient. After calculating the linear dependent coefficient, 5868 out of 7624 features remained. After redundancy reduction, 242 features were selected for the subsequent analysis. Finally, the most significant features were selected by F-test to build a prediction model by logistic regression method, including five features on T1WI, six features on CE-T1WI, four features on T2-FLAIR, and four features on ADC. For T1WI+CE-T1WI + T2-FLAIR + ADC images, eight features were selected. The selected radiomics features are listed in **Table 2**.

**Table 2:** Selected radiomics features based on T1WI, CE-T1WI, T2-FLAIR, ADC, T1WI+CE-T1WI+ T2-FLAIR + ADC model.

MRI sequences	Feature number	Selected Features	coef	relative_to_max
T1WI	5	wavelet-HHL_glcm Contrast_T1	-0.8068	-1
		lbp-2D_glszm_LoWGrayLevelZoneEmphasis_T1	-0.2824	-0.3501
		wavelet-LLH_qlszm_SmallAreaEmphasis_T1	0.2108	0.2613
		lbp-2D_glszm_GrayLevelVariance_T1	0.4451	0.5517
		lbp-3D-k_gldm_SmaliDependenceHighGrayLevelEmphasis_T1	0.5541	0.6868
CE-T1WI	6	wavelet-HLH glcm ClusterShade_CE-T1WI	0.6507	1
		lbp-3D-m2_glszm_GrayLevelNonUniformityNormalized_CE-T1WI	0.4822	0.7411
		wavelet-LHH firstorder Skewness_CE-T1WI	0.4627	0.7111
		wavelet-HHH_firstorder_Mean_CE-T1WI	-0.0938	-0.1442
		logarithm_gldm_DependenceNonUniformityNormalized_CE-T1WI	-0.4393	-0.6751
		lbp-2D_firstorder_Mean_CE-T1WI	-0.6309	-0.9695
T2-FLAIR	4	wavelet-HHH firstorder Skewness_T2	0.7012	1
		logarithm_g|cm_ClusterShade_T2	0.5346	0.7625
		lbp-2D _gIrlm_ShortRunHighGrayLevelEmphasis_T2	0.4411	0.6291
		wavelet-HLL_gldm_LargeDependenceHighGrayLevelEmphasis_T2	-0.2599	-0.3707
ADC	4	lbp-3D-k_glcm_Correlation_ADC	0.6633	1
		wavelet-LHL firstorder Mean_ADC	0.4714	0.7106
		lbp-2D_glszm_SizeZoneNonUniformity	0.0671	0.1012
		exponential firstorder90Percentile_ADC	-0.2065	-0.3113
T1WI+CE-T1WI+ T2-FLAIR + ADC	8	lbp-3D-m2_glszm_GrayLevelNonUniformityNormalized_CE-T1WI	-0.7622	-1
		wavelet-HHH firstorder Skewness_T2	-0.6573	0.8624
		lbp-2D_glszm_LoWGrayLevelZoneEmphasis_T1	-0.6396	-0.8391
		wavelet-HLH glcm ClusterShade_CE-T1WI	-0.5686	-0.746
		wavelet-HHL_glcm Contrast_T1	-0.2276	-0.2986
		lbp-2D _gIrlm_ShortRunHighGrayLevelEmphasis_T2	0.3577	0.4693
		wavelet-LHH firstorder Skewness_CE-T1WI	0.378	0.496
		lbp-3D-k_glcm_Correlation_ADC	0.568	0.7452

### Performance of the radiomics models

**Figure 3:**
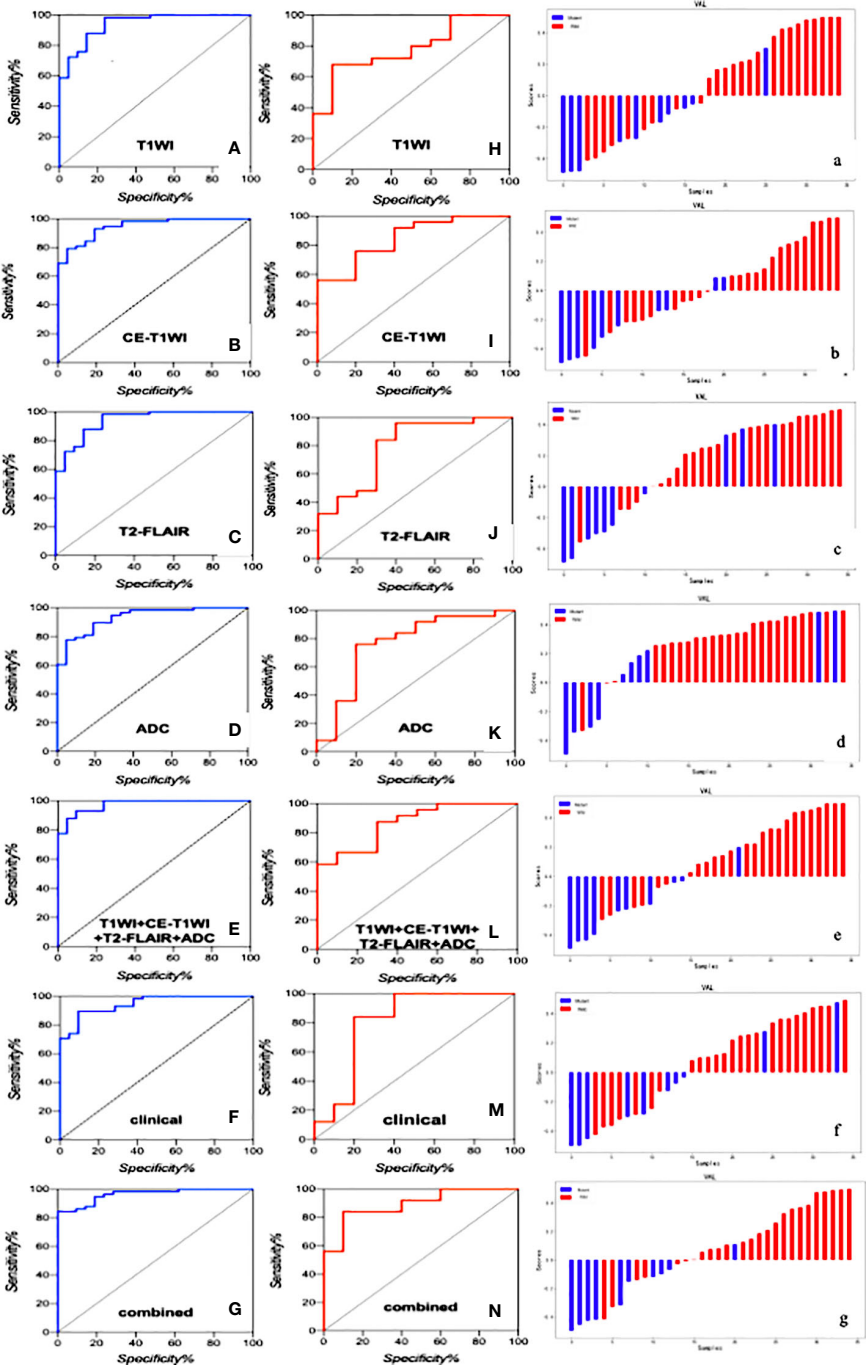
The ROC curves of the clinical model, radiomics models of T1WI, CE-T1WI, T2FLAIR, ADC, and T1WI+CE-T1WI + T2-FLAIR + ADC, and combined model in the training cohort **(A–G)** and validation cohort **(H–N)** and the waterfall plot of the validation cohort (a-g).

**Figure 4:**
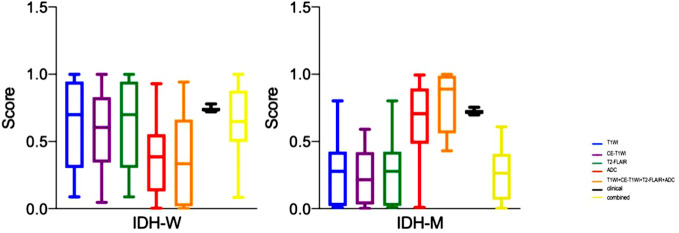
The boxplots of the clinical model, radiomics models of T1WI, CE-T1WI, T2FLAIR, ADC, and T1WI+CE-T1WI + T2-FLAIR + ADC, and combined model in the training cohort and validation cohort.

T1WI, CE-T1WI, T2FLAIR, ADC and T1WI+CE-T1WI + T2-FLAIR + ADC models produced AUC values of 0.940, 0.947, 0.947, 0.932 and 0.974 in the training cohort and 0.780, 0.848, 0.792, 0.764, 0.872 in the validation cohort. The ROC curves, waterfall plots and boxplots are shown in **Figures 3**, **4**. The accuracy, sensitivity and specificity of the radiomics models are shown in **Table 3**. The results showed that the multiparametric model of T1WI + CE-T1WI + T2-FLAIR + ADC had the best diagnostic efficacy, followed by the CE-T1WI radiomics model.

**Table 3:** The performance of the clinical model, radiomics models, and combined model.

Model	Performance	AUC	ACC	SEN	SPE
T1WI	Training	0.940(0.885-0.995)	0.861	0.862	0.857
	Validation	0.780(0.620-0.940)	0.74	0.640	0.900
CE-T1WI	Training	0.947(0.900-0.994)	0.848	0.862	0.810
	Validation	0.848(0.714-0.982)	0.729	0.560	0.800
T2-FLAIR	Training	0.947(0.900-0.993)	0.873	0.862	0.905
	Validation	0.792(0.615-0.969)	0.771	0.800	0.700
ADC T1WI+CE-T1WI +T2-FLAIR+ADC	Training	0.932(0.876-0.988)	0.823	0.809	0.828
	Validation	0.764(0.567-0.962)	0.686	0.800	0.640
	Training	0.974(0.944-1.000)	0.873	0.952	0.845
	Validation	0.872(0.742-0.992)	0.772	0.900	0.680
Clinical	Training	0.960(0.922-0.998)	0.886	0.897	0.857
	Validation	0.804(0.600-1.000)	0.729	0.885	0.600
Combined	Training	0.963(0.927-0.990)	0.861	0.879	0.810
	Validation	0.892(0.782-1.000)	0.828	0.760	0.950

### Performance of the clinico-radiological model

In the analysis of clinical data, the age of the patient and whether the tumor is intensified or not are statistically significant. In this study, these two characteristics were analyzed as a clinical model, and the AUC values of 0.960 and 0.804 were obtained for the training and validation cohorts. The clinical model was analyzed combined with the radiomics model, and the AUC values for the training and validation cohorts were 0.963 and 0.892, respectively, showing that the combined clinical-radiological model had higher diagnostic efficacy than the common radiological model. **Table 3** summarizes the sensitivity, specificity and accuracy of the clinico-radiological model. the ROC curves, waterfall plots and boxplots are shown in **Figures 3**, **4**. Among all models, the clinical–radiomics model including eight radiomics features and two clinical features achieved a performance with a classification accuracy = 0.828 and AUC = 0.892. Tests results for all variables included in the model are listed in **Supplementary Table S2**.

## Discussion

In this study, the IDH genotype of grade II-IV glioma was predicted and analyzed based on a multiparametric MRI radiomics model, and the clinical data were statistically significant in terms of patient age and whether the tumor was enhancing or not, and the clinical model and radiomics model were combined for prediction. The combined multiparametric model of T1WI + CE-T1WI + T2-FAIR + ADC had the best diagnostic performance. Thus, it can be seen that features obtained jointly from MR images of multiparametric sequences can better predict glioma IDH genotypes with higher diagnostic performancd than single sequence studies, and that combined radiomics features of multiparametric sequences can quantify comprehensive information on glioma heterogeneity. CE-T1WI contains information on local angiogenesis and blood-brain barrier disruption of the tumor. T2-FLAIR reflects the anatomical information of the tumor, and ADC provides information on the structure and density of the tumor cells. we also found that the diagnostic performance of the radiomics model of CE-T1WI was the highest in single-sequence studies, and its diagnostic performance was higher than that of ADC maps. some studies have shown that DWI sequences are not grading or stable indicator of molecular subtypes, which may be responsible for this result (29–34).

We finally extracted the most significant feature including five features on T1WI, six features on CE-T1WI, four features on T2-FLAIR, and four features on ADC. For T1WI+CE-T1WI + T2-FLAIR + ADC images, eight features were selected which included 2 first-order features and 6 texture features. The first-order features are obtained by calculating the tumor’s gray value and can respond to the tumor’s gray intensity distribution. They also capture the tumor’s heterogeneity, representing low-dimensional information easily perceived by vision. Texture features, including GLSZM, GLCM, and GLRLM, quantify the texture or tissue distribution within the tumor. These features are difficult to perceive visually but can provide information about the structure of tumor cells and the microenvironment (13, 22). In our study, we used filters to extract radiomics features from the original images. Most of the final independent imaging features comprise wavelet features, which analyze the spatial frequency changes in a comprehensive way. These features effectively capture high-frequency and low-frequency signals in the image, allowing for a detailed analysis of texture changes. The wavelet features can describe clinical problems related to the visual features of tumor images (30). Additionally, it is believed that wavelet features may contribute to our understanding of tumor morphology, pathophysiology, and proteomics (35).

In 2016, WHO classified gliomas into mutant and wild-type according to the IDH gene in the classification of central nervous system tumors (7, 8), and the IDH gene is an important genetic marker of glioma that plays an important role in glioma metabolism, pathogenesis and progression (2, 10). A growing number of studies have shown the clinical importance of genotype in developing treatment plans and assessing prognosis (20–23). Pathology is still the gold standard for diagnosis, but histological examination, as an invasive test, is invasive and has sample error, especially when stereotactic biopsy is performed. In this study, we attempted to predict IDH genotype of glioma non-invasively before surgery by the method of radiomics model based on MRI images, and provide some reference and guidance for clinical selection of surgical and postoperative radiotherapy regimens, and the results showed that this method can predict IDH genotype well.

Radiomics can dig deeper into the intrinsic features of medical images through machine learning methods and extract a large number of quantitative features that cannot be observed by the naked eye, which can support the implementation of precision medicine and individualized treatment. Some radiomics-based studies have also focused on the modeling of conventional sequences, and the obtained conventional structural sequences can reveal basic information about the tumor, such as the location, size, whether the glioma is combined with necrotic cystic lesions, the extent of edema, and the blood supply, which is helpful to provide more informed clinical information (1, 13, 23, 25). In this paper, the clinical data of the tumors were compiled in detail and statistically analyzed, in which age and whether the tumor intensified were statistically significant, indicating that age and tumor intensification were independent risk factors for predicting the IDH genotype of glioma.

Previous studies have suggested that gender is a risk factor for IDH mutation, potentially due to hormonal fluctuations in females (27, 36). However, our study found that gender did not exhibit significant predictive capability in univariate logistic regression analysis. This discrepancy underscores the need for additional research to ascertain the relevance of gender in predictive models.Although more and more scholars are using advanced techniques of MRI to analyze the relationship between IDH genotype and glioma, kim et al. (37) concluded that DWI and PWI have high diagnostic performance in predicting IDH mutations in low-grade glioma, with ADC features playing a significant role.In contrast, our study found that CE-T1WI played a more significant role, which is inconsistent with the results of this article.And in addition Park et al. (30) found that adding DTI imaging histology to conventional serial radiomics significantly improved the predictive accuracy of IDH status in low-grade glioma. However, a meta-analysis (1) revealed that despite an increasing number of scholars using more advanced examination sequences to establish feature models, the conventional MRI sequence imaging model showed better specificity in predicting IDH genotype.Niu et al. (20) found that a radiomics model based on preoperative enhanced MR was effective in predicting the IDH gene in high-grade gliomas, this is consistent with the research findings of this study, but we are not limited to a single MRI sequence. Instead, it is based on the analysis of multiple-parameter conventional MRI sequences and combined models, which are more stable and accurate. The present study was not limited to a single MRI sequence, but modeled and combined models based on multiparametric conventional MRI sequences for analysis. Tan et al. (27) studied the use of age as a clinical model to predict IDH mutations in astrocytoma based on radiomics, clinical and combined models and found that the combined model had higher diagnostic performance, this part of the research results is consistent with the findings of this study, but it only indicates the importance of age as an independent risk factor for preoperative prediction of IDH mutation in gliomas. However, this study also includes tumor enhancement as a variable in the model. Importantly, both age and tumor enhancement demonstrate satisfactory results as important variables in the model, and clinical data can be easily obtained before surgery. This is crucial for the stability of the model and its clinical application. Li et al. (17) extracted features from the enhanced, non-enhanced, necrotic, edematous, tumor core, and six regions of the whole tumor on multiparametric MRI, respectively, and showed that multiregional radiomics models can predict the mutational status of glioblastoma preoperatively. Most studies have focused on high-grade gliomas or low-grade gliomas for experimental studies (16, 18, 20, 23–25, 31, 38). The present study did not include pathological grading in the model characteristics, making the study unrestricted by pathological grading, extending the clinical application of radiomic models, and offering the possibility of preoperative prediction of IDH gene status in glioma.

This study still has some limitations. Firstly, the sample size of our study is relatively small, which may obscure the predictive value of clinical data and radiomics features of patients. This should be further investigated in larger cohorts. Secondly, we conducted a retrospective study and selected the IDH genotype for analysis. As we gather more cases, we will further investigate the relationship between abnormal expression of other important genotypes and imaging features. Lastly, our study is a single-center study, and in the future, it is necessary to collect multi-center data to validate the stability performance of the radiomics model.

## Conclusion

The multiparametric radiomics model performs better than other single sequence models in predicting the IDH genotype of gliomas. After incorporating features such as patient age and whether the tumor was enhancing or not, the results of the clinical-radiomics model are more satisfactory, indicating that the combined model is an effective tool for predicting the IDH genotype. Furthermore, the variable parameters obtained in the model contribute differently to the prediction of the IDH genotype. These findings will be beneficial for future research on using brain tumor imaging to predict molecular status and tumor invasiveness.

## Data availability statement

The original contributions presented in the study are included in the article/**Supplementary Material**. Further inquiries can be directed to the corresponding author.

## Ethics statement

The studies involving humans were approved by the ethics committee of the Aerospace Center Hospital. The studies were conducted in accordance with the local legislation and institutional requirements. Written informed consent for participation was not required from the participants or the participants’ legal guardians/next of kin in accordance with the national legislation and institutional requirements.

## Author contributions

YL: Data curation, Formal Analysis, Investigation, Writing – original draft, Writing – review & editing, Conceptualization. WL: Writing – review & editing, Supervision. DB: Methodology, Data curation, Writing – review & editing. JH: Methodology, Data curation, Writing – review & editing. ZW: Writing – review & editing, Conceptualization, Supervision.

## References

[1] Zhao J Huang Y Song Y Xie D Hu M Qiu H. Diagnostic accuracy and potential covariates for machine learning to identify IDH mutations in glioma patients: evidence from a meta-analysis. Eur Radiol (2020) 30(8):4664–74. doi: 10.1007/s00330-020-06717-9 32193643

[2] Horbinski C Berger T Packer RJ. Clinical implications of the 2021 edition of the WHO classification of central nervous system tumours. Nat Rev Neurol (2022) 18(9):515–29. doi: 10.1038/s41582-022-00679-w 35729337

[3] Stupp R Hegi ME Mason WP Bent MJ Taphoorn MJB Janzer RC. Effects of radiotherapy with concomitant and adjuvant temozolomide versus radiotherapy alone on survival in glioblastoma in a randomised phase III study: 5-year analysis of the EORTC-NCIC trial. Lancet Oncol (2009) 10(5):459–66. doi: 10.1016/S1470-2045(09)70025-7 19269895

[4] Suzuki H Aoki K Chiba K Sato Y Shiozawa Y Shiraishi Y. Mutational landscape and clonal architecture in grade II and III gliomas. Nat Genet (2015) 47(5):458–68. doi: 10.1038/ng.3273 25848751

[5] Hartmann C Hentschel B Wick W Capper D Felsberg J Simon M. Patients with IDH1 wild type anaplastic astrocytomas exhibit worse prognosis than IDH1-mutated glioblastomas, and IDH1 mutation status accounts for the unfavorable prognostic effect of higher age: implications for classification of gliomas. Acta Neuropathologica (2010) 120(6):707–18. doi: 10.1007/s00401-010-0781-z 21088844

[6] Reifenberger G Wirsching HG Knobbe-Thomsen CB. Advances in the molecular genetics of gliomas - implications for classification and therapy. Nat Rev Clin Oncol (2017) 14(7):434–52. doi: 10.1038/nrclinonc.2016.204 28031556

[7] Louis DN Perry A Reifenberger G Deimling A Figarella-Branger D Cavenee WK. The 2016 World Health Organization classification of tumors of the central nervous system: a summary. Acta Neuropathologica (2016) 131(6):803–20. doi: 10.1007/s00401-016-1545-1 27157931

[8] Komori T. The 2016 WHO classification of tumours of the central nervous system: the major points of revision. Neurologia Medico-chirurgica (2017) 57(7):301–11. doi: 10.2176/nmc.ra.2017-0010 PMC5566703 28592714

[9] Nobusawa S Watanabe T Kleihues P. IDH1 mutations as molecular signature and predictive factor of secondary glioblastomas. Clin Cancer Res (2009) 15(19):6002–7. doi: 10.1158/1078-0432.CCR-09-0715 19755387

[10] Yan H Parsons DW Jin G McLendon R Rasheed BA Yuan W. IDH1 and IDH2 mutations in gliomas. New Engl J Med (2009) 360(8):765–73. doi: 10.1056/NEJMoa0808710 PMC2820383 19228619

[11] Patel SH Bansal AG Young EB Batchala PP Patrie JT Lopes MB. Extent of surgical resection in lower-grade gliomas: differential impact based on molecular subtype. AJNR Am J Neuroradiology (2019) 40(7):1149–55. doi: 10.3174/ajnr.A6102 PMC7048539 31248860

[12] Riemenschneider MJ Jeuken JWM Wesseling P. Molecular diagnostics of gliomas: state of the art. Acta Neuropathologica (2010) 120(5):567–84. doi: 10.1007/s00401-010-0736-4 PMC2955236 20714900

[13] Gore S Chougule T Jagtap J. A review of radiomics and deep predictive modeling in glioma characterization. Acad Radiol (2021) 28(11):1599–621. doi: 10.1016/j.acra.2020.06.016 32660755

[14] Hsieh KLC Chen CY Lo CM. Radiomic model for predicting mutations in the isocitrate dehydrogenase gene in glioblastomas. Oncotarget (2017) 8(28):45888–97. doi: 10.18632/oncotarget.17585 PMC5542235 28526813

[15] Gardin I Grégoire V Gibon D Kirisli H Pasquier D Thariat J. Radiomics: Principles and radiotherapy applications. Crit Rev In Oncology/hematology (2019) 138:44–50. doi: 10.1016/j.critrevonc.2019.03.015 31092384

[16] Li Z Wang Y Yu J. Deep Learning based Radiomics (DLR) and its usage in noninvasive IDH1 prediction for low grade glioma. Sci Rep (2017) 7(1):5467. doi: 10.1038/s41598-017-05848-2 28710497 PMC5511238

[17] Li ZC Bai H Sun Q Zhao Y Lv Y Zhou J. Multiregional radiomics profiling from multiparametric MRI: Identifying an imaging predictor of IDH1 mutation status in glioblastoma. Cancer Med (2018) 7(12):5999–6009. doi: 10.1002/cam4.1863 30426720 PMC6308047

[18] Zhang X Tian Q Wang L Liu Y Li B Liang Z. Radiomics strategy for molecular subtype stratification of lower-grade glioma: detecting IDH and TP53 mutations based on multimodal MRI. J Magnetic Resonance Imaging JMRI (2018) 48(4):916–26. doi: 10.1002/jmri.25960 29394005

[19] Liu X Li Y Li S Fan X Sun Z Yang Z. IDH mutation-specific radiomic signature in lower-grade gliomas. Aging (2019) 11(2):673–96. doi: 10.18632/aging.101769 PMC6366985 30696801

[20] Niu L Feng WH Duan CF Liu YC Liu JH Liu XJ. The value of enhanced MR radiomics in estimating the IDH1 genotype in high-grade gliomas. BioMed Res Int (2020) 2020:4630218. doi: 10.1155/2020/4630218 33163535 PMC7604586

[21] Gao M Huang S Pan X Liao X Yang R Liu J. Machine learning-based radiomics predicting tumor grades and expression of multiple pathologic biomarkers in gliomas. Front In Oncol (2020) 10:1676. doi: 10.3389/fonc.2020.01676 PMC7516282 33014836

[22] Gao A Yang H Wang Y Zhao G Wang C Wang H. Radiomics for the prediction of epilepsy in patients with frontalGlioma. Front Oncol (2021) 11:725926. doi: 10.3389/fonc.2021.725926 34881174 PMC8645689

[23] Kobayashi K Miyake M Takahashi M. Observing deep radiomics for the classification of glioma grades. Sci Rep (2021) 11(1):10942. doi: 10.1038/s41598-021-90555-2 34035410 PMC8149679

[24] He A Wang P Zhu A Liu Y Chen J Liu L. Predicting IDH mutation status in low-grade gliomas based on optimal radiomic features combined with multi-sequence magnetic resonance imaging. Diagnostics (Basel Switzerland) (2022) 12(12):2995. doi: 10.3390/diagnostics12122995 36553002 PMC9776893

[25] Sun C Fan L Wang W Wang WW Liu L Duan W. Radiomics and qualitative features from multiparametric MRI predict molecular subtypes in patients with lower-grade glioma. Front In Oncol (2021) 11:756828. doi: 10.3389/fonc.2021.756828 PMC8814098 35127472

[26] Peng H Huo J Li B Cui Y Zhang H Zhang L. Predicting isocitrate dehydrogenase (IDH) mutation status in gliomas using multiparametric MRI radiomics features. J Magnetic Resonance Imaging JMRI (2021) 53(5):1399–407. doi: 10.1002/jmri.27434 33179832

[27] Tan Y Zhang ST Wei JW Dong D Wang XC Yang GQ. A radiomics nomogram may improve the prediction of IDH genotype for astrocytoma before surgery. Eur Radiol (2019) 29(7):3325–37. doi: 10.1007/s00330-019-06056-4 30972543

[28] Zhou H Chang K Bai HX Xiao B Su C Bi W. Machine learning reveals multimodal MRI patterns predictive of isocitrate dehydrogenase and 1p/19q status in diffuse low- and high-grade gliomas. J Neuro-oncology (2019) 142(2):299–307. doi: 10.1007/s11060-019-03096-0 PMC6510979 30661193

[29] Xing Z Yang X She D Lin Y Zhang Y Cao D. Noninvasive assessment of IDH mutational status in world health organization grade II and III astrocytomas using DWI and DSC-PWI combined with conventional MR imaging. AJNR. Am J Neuroradiology (2017) 38(6):1138–44. doi: 10.3174/ajnr.A5171 PMC7960080 28450436

[30] Park CJ Choi YS Park YW Ahn SS Kang SG Chang JH. Diffusion tensor imaging radiomics in lower-grade glioma: improving subtyping of isocitrate dehydrogenase mutation status. Neuroradiology (2020) 62(3):319–26. doi: 10.1007/s00234-019-02312-y 31820065

[31] Zhang B Chang K Ramkissoon S Tanguturi S Bi WL Reardon DA. Multimodal MRI features predict isocitrate dehydrogenase genotype in high-grade gliomas. Neuro-oncology (2017) 19(1):109–17. doi: 10.1093/neuonc/now121 PMC5193019 27353503

[32] Zhang B Tian J Dong D Gu D Dong Y Zhang L. Radiomics features of multiparametric MRI as novel prognostic factors in advanced nasopharyngeal carcinoma. Clin Cancer Res (2017) 23(15):4259–69. doi: 10.1158/1078-0432.CCR-16-2910 28280088

[33] Sudre CH Panovska-Griffiths J Sanverdi E Brandner S Katsaros VK Stranjalis G. Machine learning assisted DSC-MRI radiomics as a tool for glioma classification by grade and mutation status. BMC Med Inf Decision Making (2020) 20(1):149. doi: 10.1186/s12911-020-01163-5 PMC7336404 32631306

[34] Santelli L Ramondo G Della Puppa A Ermani M Scienza R d'Avella D. Diffusion-weighted imaging does not predict histological grading in meningiomas. Acta Neurochirurgica (2010) 152(8):1315–9. doi: 10.1007/s00701-010-0657-y 20428902

[35] Liang W Xu L Yang P Zhang L Wan D Huang Q. Novel nomogram for preoperative prediction of early recurrence in intrahepatic cholangiocarcinoma. Front In Oncol (2018) 8:360. doi: 10.3389/fonc.2018.00360 PMC6131601 30234019

[36] Wu S Zhang X Rui W Sheng Y Yu Y Zhang Y. A nomogram strategy for identifying the subclassification of IDH mutation and ATRX expression loss in lower-grade gliomas. Eur Radiol (2022) 32(5):3187–98. doi: 10.1007/s00330-021-08444-1 35133485

[37] Kim M Jung SY Park JE Jo Y Park SY Nam SJ. Diffusion- and perfusion-weighted MRI radiomics model may predict isocitrate dehydrogenase (IDH) mutation and tumor aggressiveness in diffuse lower grade glioma. Eur Radiol (2020) 30(4):2142–51. doi: 10.1007/s00330-019-06548-3 31828414

[38] Malik N Geraghty B Dasgupta A Maralani PJ Sandhu M Detsky J. MRI radiomics to differentiate between low grade glioma and glioblastoma peritumoral region. J Neuro-oncology (2021) 155(2):181–91. doi: 10.1007/s11060-021-03866-9 34694564

