# Supplementary material for: The value of multiparametric MRI radiomics in predicting IDH genotype in glioma before surgery
Source: Front Oncol. 2023 Nov 27;13:1265672. doi: 10.3389/fonc.2023.1265672 (PMC10711053; doi:10.3389/fonc.2023.1265672)
Supplement: Supplementary file 1 [file Table_1.docx]

| Image filter (10 types) | Explanation |
| --- | --- |
| Original | No filter applied |
| Wavelet | Wavelet filtering, yields 8 decompositions per level (all possible combinations of applying either a High or a Low pass filter in each of the three dimensions. |
| LoG | Laplacian of Gaussian filter, edge enhancement filter. Emphasizes areas of gray level change, where sigma defines how coarse the emphasised texture should be. A low sigma emphasis on fine textures (change over a short distance), where a high sigma value emphasises coarse textures (gray level change over a large distance). |
| Square | Takes the square of the image intensities and linearly scales them back to the original range. Negative values in the original image will be made negative again after application of filter. |
| SquareRoot | Takes the square root of the absolute image intensities and scales them back to original range. Negative values in the original image will be made negative again after application of filter. |
| Logarithm | Takes the logarithm of the absolute intensity + 1. Values are scaled to original range and negative original values are made negative again after application of filter. |
| Exponential | Takes the the exponential, where filtered intensity is e^(absolute intensity). Values are scaled to original range and negative original values are made negative again after application of filter. |
| Gradient | Returns the gradient magnitude. |
| LBP2D | Calculates and returns a local binary pattern applied in 2D. |
| LBP3D | Calculates and returns local binary pattern maps applied in 3D using spherical harmonics. Last returned image is the corresponding kurtosis map. |

Supplementary Table 1 10 types of image filtering methods

Supplementary Table 2 The performance of scout models in validation cohort

| Variable | AUC (95% CI) | Sensitivity | Specificity | PPV | NPV |
| --- | --- | --- | --- | --- | --- |
| \| Shape model \| \| --- \| | 0.667(0.551-0.7823) | 0.670 | 0.530 | 0.742 | 0.611 |
| \| LBP3D texture model \| \| --- \| | 0.789(0.617-0.943) | 0.640 | 0.900 | 0.941 | 0.600 |
| LBP3D texture model | 0.800(0.682-0.966) | 0.729 | 0.833 | 0.900 | 0.685 |
| \| Wavelet first order model \| \| --- \| | 0.830(0.676-0.979) | 0.920 | 0.700 | 0.852 | 0.750 |
| \| Wavelet texture model \| \| --- \| | 0.810(0.591-1.000) | 0.762 | 0.857 | 0.853 | 0.669 |
| \| Clinical model \| \| --- \| | 0.804(0.600-1.000) | 0.885 | 0.600 | 0.864 | 0.639 |
| \| Multiparametric radiomics model \| \| --- \| | 0.872(0.742-0.992) | 0.900 | 0.680 | 0.950 | 0.600 |
| Clinical-radiomics model | 0.892(0.782-1.000) | 0.760 | 0.950 | 0.923 | 0.701 |
